# Rest Intolerance, Emotional Distress, Insomnia, and Adaptive Coping Strategies: A Validation and Serial Mediation Analysis Study

**DOI:** 10.1007/s11126-025-10176-0

**Published:** 2025-06-24

**Authors:** Mehmet Avcı

**Affiliations:** Faculty of Education, Guidance and Psychological Counseling Program, Recep Tayyip Erdogan University, Rize, 53200 Turkey

**Keywords:** Rest intolerance, Self-compassion, Emotional distress, Resilience, Insomnia

## Abstract

Rest intolerance, characterized by negative emotions like shame and guilt during periods of rest, has become an increasingly prevalent health concern. This inability to relax or disengage from activity can exacerbate psychopathology by contributing to stress and anxiety, underscoring the need for further investigation. This study aimed to assess the reliability and validity of the Turkish version of the Rest Intolerance Scale (RIS) and to investigate the mediating role of self-compassion and resilient mindset in the relationship between rest intolerance, emotional distress, and sleep complaints among Turkish young adults. A total of 475 young adults participated, with 349 females (73.5%) and 126 males (25.5%) aged 18 to 28 years. The results confirmed the RIS’s reliability and validity in identifying individuals’ discomfort or symptoms during rest. Rest intolerance was found to be a significant predictor of insomnia complaints and emotional distress. Additionally, rest intolerance negatively affected self-compassion and resilient mindset, while self-compassion mediated the relationship between rest intolerance and both insomnia complaints and emotional distress. These findings highlight the importance of addressing rest intolerance as a key factor in promoting mental health and well-being. Self-compassion emerged as a crucial element in reducing the negative effects of rest intolerance, suggesting its potential as an intervention target.

## Introduction

In today’s fast-paced, always-connected world, many individuals find it increasingly difficult to relax and fully unwind, even when there is an opportunity to do. This trend has led to the emergence of the concept “rest intolerance” that has, in recent years, gained widespread attention in psychology. Rest intolerance refers to the feelings of guilt, shame, and other negative emotions experienced by individuals when they are attempting to rest [1–3]. For instance, Koo [4] defined the phenomenon of rest intolerance as “leisure guilt,” characterizing it as the experience of guilt or distress associated with spending time on leisure rather than work, with guilt being the predominant emotion. It has also been emphasized that individuals with a propensity to experience guilt, distress, or anxiety while engaging in leisure activities are more likely to prioritize work over relaxation. Research findings suggest that individuals with elevated levels of leisure guilt tend to allocate more time to work-related tasks [2, 4], potentially leading to workaholism and increasing the risk of burnout.

However, rest intolerance also encompasses other negative emotions, such as remorse, distress, and anxiety, as well as obsessive-compulsive tendencies frequently exhibited by individuals who frequently over-focus on work during their leisure time [2, 5].

## Emotional Distress and Sleep Complaints

In a high-pressure academic environment, students frequently experience emotional distress characterized by elevated levels of stress, anxiety, and burnout. All these feelings can adversely affect students’ mental well-being and academic performance [6, 7]. Furthermore, the lack of adequate rest and sleep problems can exacerbate these challenges, resulting in a further decline in psychological health and academic outcomes [4]. Particularly common symptoms showed by university students are insomnia and sleep complaints [8, 9], both of which can stem from rest intolerance. Available research documented a reciprocal relationship between insomnia and anxiety, with each condition exacerbating the other [10, 11]. Previous studies also demonstrated that insomnia exerts a causal influence on anxiety, suggesting that the characteristics of insomnia should be integrated into strategies for anxiety prevention and intervention [12]. Other lifestyle factors that can contribute to students’ delaying their sleep include academic pressures, part-time employment, social activities, and recreational pursuits, potentially resulting in irregular sleep patterns or insomnia [13].

Poor sleep quality may also exacerbate emotional difficulties through impairments in executive functioning, including deficits in memory, attention, multitasking, and task completion [14, 15]. The emotional burden of experiencing guilt during leisure time may elevate stress levels and contribute to emotional distress. Inability to fully relax due to feelings of guilt compromises individuals’ capacity for mental and emotional recovery, which may exacerbate symptoms of anxiety or sadness [16]. Furthermore, the presence of leisure guilt may discourage engagement in future leisure activities, as individuals start associating relaxation with negative emotions. This avoidance leads to heightened burnout, diminished life satisfaction, and an overall sense of emotional depletion [17]. A potential way to counteract these negative effects is through self-compassion and a resilient mindset, both of which were reported to enhance well-being [18].

### Self-Compassion and Resilient Mindset

Self-compassion is the practice of treating oneself with kindness during times of suffering [19]. The psychological construct of self-compassion serves as a critical buffer against negative self-evaluations and distress [20]. Self-compassion was documented to be associated with improved sleep quality through several mechanisms, including the promotion of positive coping strategies, reduction of rumination, and potential buffering effects against hyperarousal and stress [21]. The results of numerous studies confirmed that individuals with high levels of self-compassion tend to exhibit lower levels of anxiety and depression and higher levels of resilience [22, 23]. By contrast, rest intolerance, which is associated with the difficulty relaxing or recovering during rest periods, can exacerbate sleep problems and contribute to a decrease in self-compassion. This, in turn, can heighten emotional distress, making it more difficult for individuals to manage stress effectively. Furthermore, self-compassion practices were reported to reduce emotional distress and facilitate more adaptive coping mechanisms in challenging situations [24, 25], thus offering a potential solution to counteract the negative effects of rest intolerance. Similarly, resilience is generally assumed to be characterized by recovering from adversity, adapting to difficult circumstances, and maintaining a positive outlook [26]. Available evidence indicates that resilient individuals are better equipped to cope with stressors and thus exhibit lower levels of anxiety and emotional distress in response to challenges [27]. In recent years, interest in fostering resilience through interventions promoting adaptive thinking patterns has considerably increased, as such approaches help individuals to develop a more robust psychological framework [28]. For instance, a recent meta-analysis found that, as a universal prevention strategy, resilience interventions are effective in supporting higher education students [29].

### The Present Study

In today’s society, the strong emphasis on competition and productivity adversely affects individuals’ attitudes toward rest, frequently leading to anxiety surrounding relaxation and a diminished sense of its value [2]. Such circumstances can create a toxic mindset where rest is viewed as a luxury, rather than a necessity. Accordingly, many students believe that success depends on their ability to constantly work and perform, which further perpetuates a vicious cycle of stress and exhaustion. Among studentships, rest is frequently undervalued and seen as an indulgence. In university settings characterized by relentless pressure, students tend to grapple with these feelings, which may result in significant psychological and physiological issues such as sleep disturbances and emotional distress. In Turkish culture, the combination of stringent academic demands, social obligations, and challenges associated with newfound independence frequently compels university students to prioritize productivity over well-being. Furthermore, the requirement to take exams to secure employment post-graduation exacerbates academic pressure, adding yet another layer of stress to students’ already demanding workloads. In this context, rest intolerance emerges as a critical issue, as students face challenges in balancing rest, study, and socializing periods, which ultimately adversely impacts their mental and physical well-being.

**Fig. 1:**
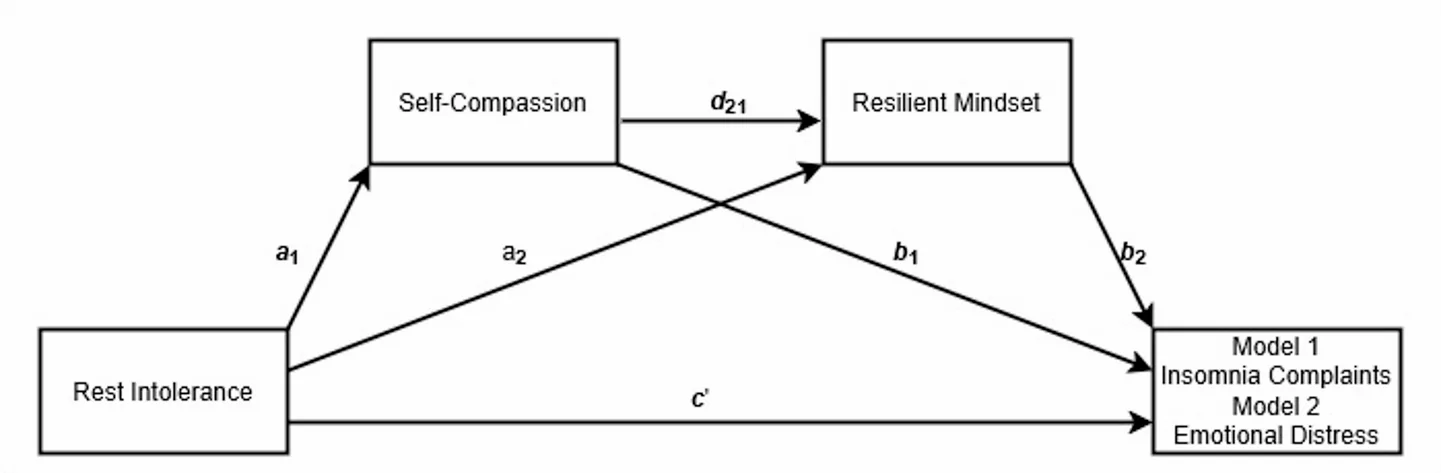
Proposed serial mediation model

Accordingly, the present study has the following two primary objectives. First, we aim to adapt the novel psychological construct RIS to Turkish university students. Our second goal is to explore the impact of rest intolerance and its potential associations with self-compassion, resilience, emotional distress, and sleep quality. A detailed representation of the proposed model can be seen in Fig. 1. Based on our review of previous research, the following four hypotheses are formulated:

**H1**– The adaptation of the Rest Intolerance Scale will exhibit sufficient reliability and validity to evaluate rest intolerance among Turkish university students.

**H2**– Rest intolerance will positively correlate with insomnia complaints and emotional distress, with individuals experiencing higher levels of rest intolerance being more likely to report sleep disturbances and emotional distress.

**H3**– Higher levels of rest intolerance will negatively correlate with self-compassion and resilient mindset, suggesting that individuals with greater rest intolerance exhibit lower self-compassion and resilience.

**H4**– Self-compassion and a resilient mindset will mediate the relationship between rest intolerance and emotional distress and insomnia complaints, thus buffering the negative effects of rest intolerance (Fig. 1).

## Method

### Participants

In this scale adaptation and mediation study, the data were collected through an online survey from a total of 475 university students in Türkiye. Data collection was conducted between October 2024 and February 2025. A G*Power analysis was conducted to determine the minimum required sample size for testing a mediation model with five predictors. The following parameters were specified for the analysis: an effect size of 0.15, an alpha level (significance threshold) of 0.05, and a desired power of 0.80. The results indicated that a minimum sample size of 119 participants must be used to detect the specified effect size with adequate statistical power. For the current study, a total sample size of 475 participants was obtained, which exceeds the required size of 119. The participants’ ages ranged from 18 to 28 years old, with a median age of 21 and a mean age of 21.17 (SD = 1.78). In terms of gender distribution, 73.5% of the participants were female, while 26.5% were male. Most participants (56.6%) resided in dorms, followed by those living at home with family (35.8%). The remaining participants were distributed among those living at home with friends (4.0%) and those living alone (3.6%).

### Measures

#### Rest Intolerance Scale (RIS)

Rest Intolerance, a 24-item self-report scale [2], was used to (1) adapt to Turkish and (2) to measure students’ perceptions towards rest (e.g., When I’m resting or having fun, I worry about being passed by someone else”, “I think rest periods should be kept as short as possible). The scale is scored on a 5-point Likert scale, ranging from 1 = “strongly disagree” to 5 = “strongly agree,” with higher total scores on the scale indicating more severe rest intolerance. The scale consists of four dimensions—namely, cognitive bias, obsessive thinking, social comparison, and negative feelings. In a previous study, Cronbach’s alpha coefficients of the aforementioned dimensions were 0.69, 0.89, 0.93, and 0.95, respectively [2]. The psychometric properties of the Turkish version of the RIS are reported in the Results section of the present paper.

#### The Self-Compassion Scale-Short Form (SCS-SF)

The SCS-SF was developed by Raes et al. [30] as a tool to evaluate the frequency with which individuals exhibit kindness and care towards themselves during challenging circumstances. The scale consists of 12 items (e.g., “When I fail at something that is important to me, I experience a sense of inadequacy”), to be rated on a 5-point Likert scale (from 0 = “almost never” to 5 = “almost always”). Higher scores on the scale correspond to higher levels of self-compassion. In a previous Turkish validation study, the scale demonstrated adequate internal consistency, with a Cronbach’s alpha amounting to 0.83 [20]. In the present study, the internal reliability estimate was 0.75.

#### Emotional Distress Scale (EDS)

The 8-item EDS [20] was employed to evaluate the participants’ levels of emotional distress (e.g., “Difficulty in relaxing and calming yourself”). The study participants rated their agreement with each statement on a 5-point Likert scale, ranging from 0 = “never” to 4 = “extremely.” Total scores were derived by summing the ratings across all eight items, with higher scores reflecting a greater emotional distress. In the development study, the EDS demonstrated strong internal consistency, with a Cronbach’s alpha of 0.88 [20]. In the present study, internal consistency of the scale was 0.89.

#### Resilient Mindset Scale (RMS)

The RMS evaluates the degree to which individuals’ cognitive processes are characterized by consciousness, flexibility, realism, and creativity [31]. The scale comprises six items (e.g., “Even in stressful situations, I can observe what is happening with an open mind”), with responses measured on a 5-point Likert scale ranging from 0 = “Almost never true” to 4 = “Almost always true.” Internal consistency of this scale was reported to be 0.86 in the development study [31]; in the present study, this value amounted to 0.91.

#### The Basic Scale of Insomnia Complaints and Sleep Quality (BASIQS)

The BASIQS was developed by Allen Gomes et al. [32] and adapted to Turkish by Mıhçıoğlu et al. [33]. This scale consists of 7 items and two dimensions: difficulty falling asleep and difficulty waking up. These dimensions evaluate the factors such as duration of time required to fall asleep, frequency of difficulties initiating sleep, frequency of nighttime awakenings, occurrence of waking up early in the morning or late at night, subjective perception of sleep quality regardless of its duration, and overall quality of sleep (e.g., “How often do you have difficulty falling asleep?”). The scores for each item range from 0 to 4, with higher scores indicating greater levels of sleep complaints. Internal consistency of this scale was reported as 0.75 in the adaptation study [33], and 0.70 for the present study.

### Study Procedure

First, we obtained permission to use and adapt the RIS into Turkish from the authors of the scale. Furthermore, on receiving ethical approval from the author’s institution (2024/344), the scale was translated into Turkish in full compliance with the guidelines recommended by the International Test Commission [34]. The survey was administered using an online platform (Google Forms), with an average completion time of approximately 10–15 min per participant, and no incentives were provided for participation. At the beginning of the survey, the participants were informed about the scope of the study, provided their informed consent, and participated voluntarily.

### Translation Process

In the adaptation of the RIS into Turkish, the original English version of the scale was first translated into Turkish by three academicians proficient in English and an English language instructor. Subsequently, the Turkish version was back-translated, and its consistency with the original English version was evaluated. Next, a Turkish Language Teaching Department faculty member reviewed the Turkish form for meaning and grammatical accuracy, and all necessary revisions were made to finalize the Turkish version. In the final stage, the form was evaluated by three faculty members specialized in counseling, measurement, and evaluation who identified unclear expressions with multiple meanings; all unclear items were adjusted based on this feedback.

### Data Analysis

The SPSS (version 27) statistical package program and JASP 0.19.1.0 were used to run all basic statistical analyses (frequency analysis, mean, standard deviation, skewness, kurtosis, correlation, and reliability) and network structure between survey items. Skewness-kurtosis values were examined to determine the distribution of the data and values in the range of -2 and + 2 indicate that scores were normally distributed [35]. As the first step in adapting the scale, Exploratory Factor Analysis (EFA) was conducted using the Principal Component Method with Varimax rotation to examine construct validity and determine the factor structure. The reliability of the scales used in the study was assessed through reliability testing. To establish the validity of the scale, Confirmatory Factor Analysis (CFA), one of the Structural Equation Modeling (SEM) techniques, was performed. IBM SPSS Amos 21 Graphics was used to test the fit of the models on all scales. Finally, the PROCESS macro for SPSS (Model 6) [36] was used to determine the mediation effects on the relationship between the independent and the dependent variable. A p-value of < 0.05 was considered statistically significant.

## Results

### Psychometric Characteristics of the Turkish Version of the RIS

First, an Exploratory Factor Analysis (EFA) was conducted for the validity analysis of the RIS. Before performing the exploratory factor analysis, the Kaiser-Meyer-Olkin (KMO) test was used to assess the adequacy of the sample size for factor analysis. The KMO value was found to be 0.9556, indicating that the sample size was highly sufficient for factor analysis. The Bartlett’s Test of Sphericity was performed to determine whether the data matrix was an identity matrix and whether the correlation between variables was sufficient for factor analysis, in other words, whether the data structure was suitable for factor analysis. The results confirmed that the dataset was appropriate for factor analysis (X² = 82339.222, *p* < 0.001).

**Fig. 2:**
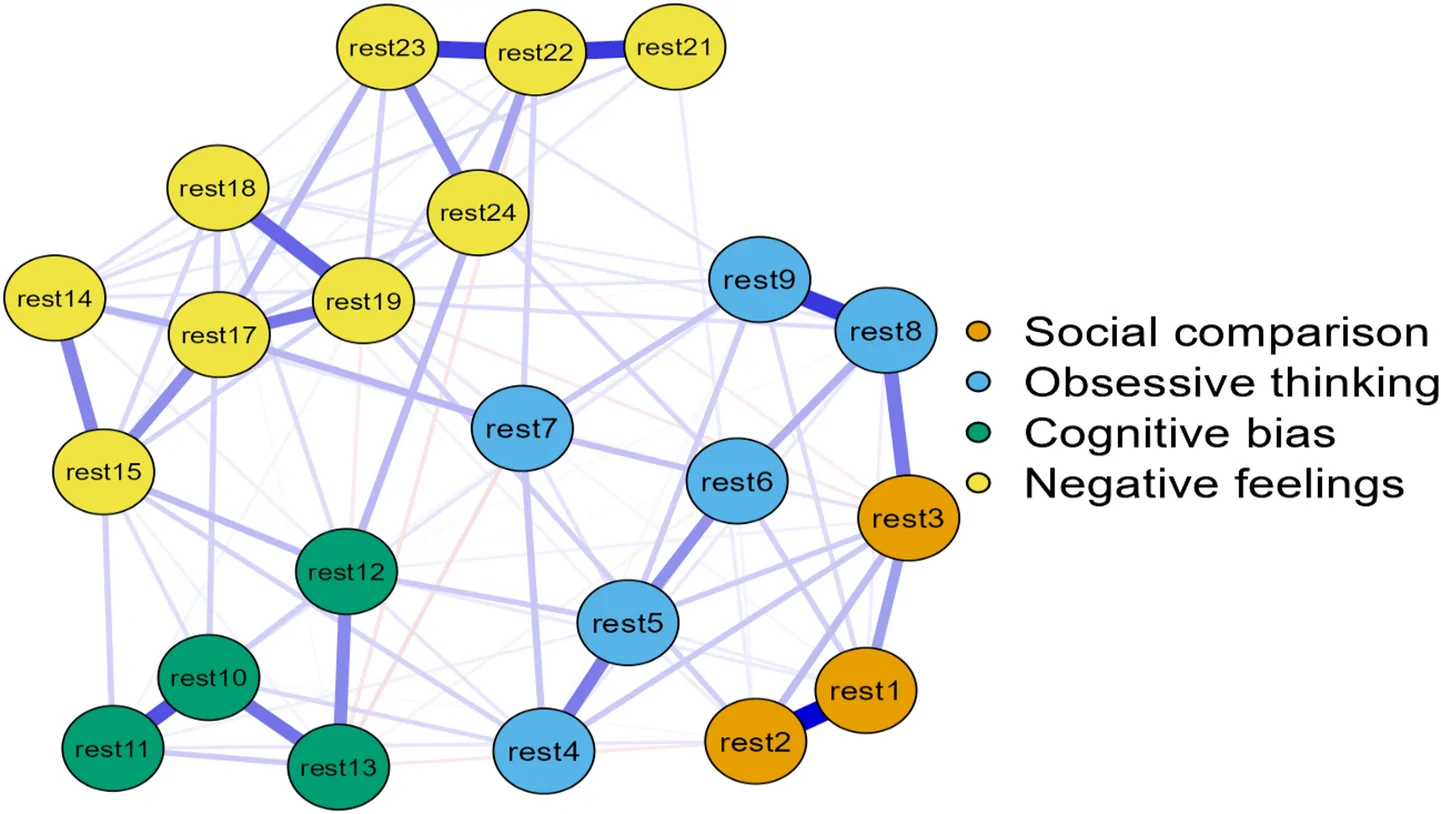
Turkish version of rest intolerance scale-graphical network

To reveal the factor structure of the RIS, the Principal Component Analysis (PCA) was used as the factor extraction method, and Varimax rotation was applied. In the EFA, a factor loading threshold of 0.300 was set. In the four-factor solution, no items were found with a common variance below 30%. Items 16 and 20 were identified as complex items, as the difference between their factor loadings was less than 0.100, and they were removed from the scale. The factor loadings ranged between 0.554 and 0.862, indicating a highly adequate level. After the Varimax rotation, the scale items were grouped under four factors, (Social Comparison, Obsessive thinking, Cognitive bias, Negative feelings) which together explained 71.604% of the total variance. This finding suggests that each identified factor contributes sufficiently to the total variance. Furthermore, we conducted a network analysis to provide a more detailed examination of the factor structures (Fig. 2). In the network graph, each node represents one of the 22 items included in the scale, visually illustrating the individual components of the construct being measured. The edges, or lines connecting the nodes, indicate the statistical associations or interactions between the items. These connections are typically weighted, with the thickness of each edge reflecting the strength of the relationship between the corresponding items—thicker edges denote stronger associations. The results of the network analysis provide empirical support for the four-dimensional structure of the Turkish version of the Rest Intolerance Scale (RIS), as the items naturally cluster into distinct yet interconnected dimensions, consistent with the theoretical model underlying the scale.

In addition, model fit indices were used to evaluate how well the structural model fits the observed data. Results indicated that the chi-square/degrees of freedom ratio (χ²/df), Root Mean Square Error of Approximation (RMSEA), Goodness-of-Fit Index (GFI), Adjusted Goodness-of-Fit Index (AGFI), Comparative Fit Index (CFI), Normed Fit Index (NFI), Tucker–Lewis Index (TLI), and Standardized Root Mean Square Residual (SRMR) values all fall within the acceptable fit range, suggesting that the model demonstrates a reasonably good fit to the data (Table 1). The internal consistency of the RIS Turkish form was assessed. The analysis revealed strong internal consistency, with Cronbach’s alpha coefficients of α = 0.85 for social comparison, 0.90 for obsessive thinking, 0.81 for cognitive bias, 0.94 for negative feelings, and 0.95 for the total score. These results provide evidence that the 22-item Turkish form of the RIS possesses adequate psychometric properties for use with Turkish university students.

**Table 1:** Goodness-of-fit values of the structural model

	Structural Model Values	Perfect fit	Acceptable fit
χ2/df	3.858	0 ≤ χ^2^/sd ≤ 2	0 ≤ χ^2^/sd ≤ 5
RMSEA	0.078	0 ≤ RMSEA ≤ 0.05	0.05 < RMSEA ≤ 0.10
GFI	0.900	0.95 ≤ GFI ≤ 1.00	0.90 ≤ GFI ≤ 0.95
AGFI	0.850	0.95 ≤ AGFI ≤ 1.00	0.85 ≤ AGFI ≤ 0.90
CFI	0.931	0.95 ≤ CFI ≤ 1.00	0.90 ≤ CFI < 0.95
NFI	0.909	0.95 ≤ NFI ≤ 1.00	0.90 ≤ NFI ≤ 0.95
TLI	0.919	0.95 ≤ TLI ≤ 1.00	0.90 ≤ TLI < 0.95
SRMR	0.051	0 ≤ SRMR ≤ 0.05	0.05 < SRMR ≤ 0.08

Note. Model fit indices were produced based on Hu & Bentler [37]; Kline [38]; Tabachnick & Fidell [39]

### Descriptive and Correlation Analyses

On determining reliability and running confirmatory factor analyses, descriptive characteristics of the study variables were examined (Table 2). The findings showed sufficient reliability coefficients ranging from 0.650 to 0.915. The RIS scale had a median score of 46 (range: 22–110) and a mean of 49.56 ± 21.1. The SCS-SF median score was 39 (range: 16–60), with a mean of 39.97 ± 7.77. The RM scale had a median of 15 (range: 0–24) with a mean of 14.65 ± 6, while the ED scale showed a median of 10 (range: 0–32) and a mean of 11.69 ± 7.43. The Insomnia Complaints and Quality of Sleep scale had a median of 13 (range: 2–27) with a mean of 12.84 ± 4.52. In addition, the results of the bivariate correlation analysis indicated that RIS exhibited significant positive correlations with Insomnia Complaints and ED, and significant negative correlations with self-compassion and a resilient mindset.

**Table 2:** Descriptive statistics and correlation analyses of study variables

	Mean$\:\pm\:$SD	$\:\boldsymbol{\alpha\:}$	RIS	SCS-SF	RM	ED	IC
RIS	49.56 ± 21.1	0.956	1				
SCS-SF	39.97 ± 7.77	0.758	− 0.405**	1			
RM	14.65 ± 6	0.915	− 0.212**	0.519**	1		
ED	11.69 ± 7.43	0.899	0.408**	− 0.414**	− 0.160**	1	
IC	12.84 ± 4.52	0.650	0.143**	− 0.200**	− 0.146**	0.347**	1

Note. *N* = 475, *p < 0.05, **p < 0.01, SD: Standard deviation, RIS: Rest Intolerance Scale, SCS-SF: Self-compassion Scale-Short Form, RM: Resilient Mindset, ED: Emotional Distress, IC: Insomnia Complaints

### Serial Mediation Models

Mediation analyses were conducted using the PROCESS macro for SPSS (Model 6) [36] to examine the mediation effects of SCS-SF and RM on the relationship between RIS (independent variable) and ED, IC (dependent variable). The statistical significance of the mediator variables was tested using 5000 bootstrap samples. In this method, a 95% confidence interval that does not include zero is assumed to indicate a significant indirect effect [40].

The first analysis was conducted to examine whether SCS-SF and RM mediate the relationship between RIS and IC (Table 3). Examining the first-stage relationships, the effect of RIS on SCS-SF (a1) was significant (b = -0.149, *p* = 0.000), meaning that a one-unit increase in RIS leads to an average decrease of 0.149 in SCS-SF. By contrast, the relationship between RIS and RM (a2) was not significant (b = -0.001, *p* = 0.956), suggesting that RIS does not have a meaningful effect on RM. With regard to the second-stage mediations, the effect of SCS-SF on IC (b1) was significant (b = -0.081, *p* = 0.013), indicating that a one-unit increase in SCS-SF leads to an average decrease of 0.081 in IC. However, the effect of RM on IC (b2) was not significant (b = -0.044, *p* = 0.270), meaning that RM does not significantly contribute to IC. In terms of overall effects, the total effect of RIS on IC (c) was significant (b = 0.031, *p* = 0.002), indicating that RIS influences IC before considering the mediators. However, the direct effect of RIS on IC (c’) was not significant (b = 0.016, *p* = 0.131), suggesting that RIS does not directly impact IC when accounting for SCS-SF and RM. The indirect effect of RIS on ICQS through SCS-SF (a1 * b1) was significant (b = 0.012, CI [0.001, 0.024]), confirming that SCS-SF acts as a mediator. By contrast, the indirect effect through RM (a1 * b2) was not significant (b = 0.000, CI [-0.002, 0.002]), and so was the sequential mediation pathway (a1 * d21 * b1) (b = 0.003, CI [-0.002, 0.008]).

Taken together, these results indicate that SCS-SF significantly mediates the relationship between RIS and IC, whereas RM does not serve as a mediator. Since the direct effect of RIS on IC was not significant after accounting for mediators, full mediation occurs through SCS-SF. In summary, RIS influences IC indirectly through SCS-SF, while RM does not play a significant role in this mediation process.

**Table 3:** Results of serial mediation analysis for model 1

Effects	Coefficient	SE	t	*p*	95% CI (LL-UL)
Constant	15.934	1.452	10.973	< 0.001*	(13.080, 18.787)
Rest$\:\to\:$SCS-SF (a_1_)	-0.149	0.015	-9.627	< 0.001*	(-0.180, -0.119)
Rest$\:\to\:$RM (a_2_)	-0.001	0.012	-0.056	0.956	(-0.025, 0.023)
SCS-SF$\:\to\:$IC (b_1_)	-0.081	0.033	-2.480	0.013*	(-0.145, -0.017)
RM$\:\to\:$IC (b_2_)	-0.044	0.040	-1.105	0.270	(-0.122, 0.034)
Rest$\:\to\:$IC (total effect) (c)	0.031	0.010	3.147	0.002*	(0.012, 0.050)
Rest$\:\to\:$IC (c’) (direct effect)	0.016	0.011	1.513	0.131	(-0.005, 0.037)
SCS-SF$\:\to\:$RM (d_21_)	0.400	0.033	12.049	< 0.001*	(0.335, 0.476)
Inderect effect (a_1_*b_1_)	0.012	0.006	-	-	(0.001, 0.024)
Inderect effect (a_1_*b_2_)	0.000	0.001	-	-	(-0.002, 0.002)
Inderect effect (a_1_*d_21_*b_1_)	0.003	0.003	-	-	(-0.002, 0.008)
*R: 0.217*,* R*^*2*^:*0.047*,* F = 7.751*,* p = < 0.001*					

Note. *p < 0.05, SE: Standard Error, LL: Lower Level, UP: Upper Level, CI: Confidence Interval. RIS: Rest Intolerance Scale, SCS-SF: Self-compassion Scale-Short Form, RM: Resilient Mindset, ED: Emotional Distress, IC: Insomnia Complaints

The second analysis was conducted to examine whether SCS-SF and RM mediate the relationship between RIS and ED (Table 4). In terms of the first-stage relationships, the effect of RIS on SCS-SF (a1) was significant (b = -0.149, *p* = 0.000), while the relationship between RIS and RM (a2) was not significant (b = -0.001, *p* = 0.956), suggesting that RIS does not have a meaningful effect on RM. Regarding the second-stage mediations, the effect of SCS-SF on ED (b1) was significant (b = -0.322, *p* = 0.000). However, the effect of RM on ED (b2) did not reach statistical significance (b = 0.094, *p* = 0.104), meaning that RM does not significantly contribute to ED. In terms of overall effects, the total effect of REST on ED (c) was significant (b = 0.144, *p* = 0.000), indicating that RIS influences ED before considering the mediators. The direct effect of RIS on ED (c’) also remained significant (b = 0.101, *p* = 0.000), suggesting that RIS has a direct impact on ES in addition to any mediation pathways. The indirect effect of RIS on ED through SCS-SF (a1 * b1) was significant (b = 0.136, CI [0.087, 0.188]), confirming that SCS-SF acts as a mediator. However, the indirect effect through RM (a1 * b2) was not significant (b = 0.000, CI [-0.010, 0.010]), and so was the sequential mediation pathway (a1 * d21 * b1) (b = -0.016, CI [-0.040, 0.007]).

The aforementioned results indicate that SCS-SF significantly mediates the relationship between RIS and ED, whereas RM does not serve as a mediator. Since the direct effect of RIS on ED was still significant after accounting for the mediators, this suggests partial mediation rather than full mediation. In summary, we found that RIS influences ED both directly and indirectly through SCS-SF, while RM did not play a significant role in this mediation process. The overall model was statistically significant (F = 50.866, *p* = 0.000) and explained 24.5% (R² = 0.245) of the variance in ED.

**Table 4:** Results of serial mediation analysis for model 2

Effects	Coefficient	SE	t	*p*	95% CI (LL-UL)
Constant	18.163	2.124	8.551	< 0.001*	(13.989, 22.336)
Rest$\:\to\:$SCS-SF (a_1_)	-0.149	0.015	-9.627	< 0.001*	(-0.180, -0.119)
Rest$\:\to\:$RM (a_2_)	-0.001	0.012	-0.056	0.956	(-0.025, 0.023)
SCS-SF$\:\to\:$ES (b_1_)	-0.322	0.048	-6.729	< 0.001*	(-0.416, -0.228)
RM$\:\to\:$ED (b_2_)	0.094	0.058	1.628	0.104	(-0.020, 0.208)
Rest$\:\to\:$ED (total effect) (c)	0.144	0.015	9.720	< 0.001*	(0.115, 0.173)
Rest$\:\to\:$ED(c’) (direct effect)	0.101	0.015	6.573	< 0.001*	(0.071, 0.132)
SCS-SF$\:\to\:$RM (d_21_)	0.400	0.033	12.049	< 0.001*	(0.335, 0.465)
Inderect effect (a_1_*b_1_)	0.136	0.026	-	-	(0.087, 0.188)
Inderect effect (a_1_*b_2_)	0.000	0.005	-	-	(-0.010, 0.010)
Inderect effect (a_1_*d_21_*b_1_)	-0.016	0.012	-	-	(-0.040, 0.007)
*R: 0.495*,* R*^*2*^:*0.245*,* F = 50.866*,* p = < 0.001*					

Note. *p < 0.05, SE: Standard Error, LL: Lower Level, UP: Upper Level, CI: Confidence Interval

## Discussion

In this study, we examined the relationship between rest intolerance, on the one hand, and insomnia complaints and emotional distress, on the other hand. We also determined whether self-compassion and resilient mindset mediated this relationship. Our results revealed that, in line with H2, Turkish students’ rest intolerance positively correlated with sleep problems and emotional distress, supporting our expectation that rest intolerance is a factor leading to insomnia and distress. While previous research examined similar concepts, such as leisure guilt as a predictor of poorer health-related factors (e.g., higher anxiety, increased stress, decreased well-being [4]; our study is the first to investigate the predictor of insomnia and emotional distress within the context of rest intolerance. Wang et al. [2] identified the psychological connotations of rest intolerance as including negative feelings, social comparison, obsessive thinking, and cognitive bias. Among these feelings, anxiety, guilt, shame, and other negative evaluations were described as the most significant indicators of rest intolerance. Prior research also elucidated that stress could act as a catalyst for anxiety [41], which, in turn, significantly impairs sleep quality [42]. Our findings highlight the critical role of rest intolerance as a strong predictor of both sleep disturbances and emotional distress, emphasizing how inability to rest comfortably may exacerbate anxiety and further compromise sleep quality.

In the context of the collectivistic Turkish culture that emphasizes social conformity, academic achievement, and professional success, students frequently experience pressure to remain industrious and engaged [43]. This pressure is aggravated by the highly competitive nature of the Turkish education system, which prioritizes success on standardized exams and academic performance as key markers of individual worth. Moreover, the cultural expectation of constant self-improvement, coupled with the pressure to demonstrate academic competence, further exacerbates the challenge of achieving a healthy work-life balance [44]. In this context, rest intolerance among Turkish university students can represent not only a personal issue, but also reflect the cultural and societal values that prioritize productivity over well-being.

### Rest Intolerance, Self-compassion, and Resilient Mindset

The results of this study indicate that rest intolerance is significantly and negatively associated with both self-compassion and a resilient mindset, individually (H3). Specifically, we found that students with higher rest intolerance may be less likely to treat themselves kindly during difficult times or show themselves understanding when experiencing stress or setbacks. There might also be a reciprocal relationship that individuals who practice more self-compassion may feel more deserving of rest and recovery [19, 25]. Self-compassion might help to reduce the feelings of guilt or discomfort associated with taking time off, potentially making it easier to relax and recharge [20, 30]. This, in turn, could further reduce rest intolerance. In addition, we also found that higher levels of rest intolerance are associated with lower levels of resilient mindset. Overall, such mindset involves facing challenges with strength, transforming adversity into growth, and maintaining a positive outlook, supported by mental toughness, mindfulness, and hope [5, 31]. By contrast, rest intolerance, which impedes relaxation and recovery [2], can hinder students’ ability to fully engage with challenges and stifle resilience, making it more difficult to adapt and bounce back from adversity.

Regarding the results of our mediation analysis, rest intolerance was found to indirectly affect emotional distress and sleep quality through its impact on self-compassion (H4). Said differently, individuals who experience higher levels of rest intolerance are likely to have lower self-compassion, which, in turn, leads to greater emotional distress and poorer sleep quality. Although resilient mindset was related to emotional distress and sleep complaints, its contribution to serial mediation was found to be non-significant. The importance of self-compassion on increasing students’ well-being [45], reducing psychological and emotional distress [46–48], and improving sleep quality [21, 49, 50]; is well documented in the literature. Specifically, previous research conducted among Turkish university students revealed a significant relationship between self-compassion and various psychological outcomes, including psychological well-being, distress, resilience, anxiety, and social safety [47, 51]. Consistent with prior research, we also found that students with higher levels of self-compassion experience lower levels of rest intolerance, which subsequently leads to a reduction in sleep complaints and emotional distress.

Regarding resilient mindset, cognitive bias could result in faulty perceptions of rest, where individuals may view rest as unearned or wrong [52]. In that case, even if an individual has a resilient mindset, their altered perception of rest might cause them to have negative evaluations of themselves and their behavior during rest [2]. This could create a cycle where resilience alone cannot overcome the cognitive distortions associated with rest intolerance. In addition, there is evidence to indicate that rest intolerance is linked to social comparison [2], which could be another reason why we did not find that resilient mindset can significantly mediate the relationship. That is, if individuals are constantly comparing their rest to that of others, it could create a competitive atmosphere that exacerbates feelings of inadequacy. Even with a resilient mindset, the constant pressure of comparison might make it difficult to rest effectively and aggravate emotional distress or worry [30, 53]. This could make resilience less impactful in these situations, as external factors (e.g., social comparison) play a stronger role than internal coping resources.

### Implications and Limitations

The findings of this study provide several important implications for both academic and practical interventions aimed at improving university students’ well-being, particularly in the context of rest intolerance. First, the significant positive correlation between rest intolerance, sleep complaints, and emotional distress emphasizes the critical need to address rest intolerance as a distinct psychological factor that contributes to poor health outcomes, such as insomnia and increased distress. Our results underscore the importance of developing interventions to mitigate rest intolerance, potentially through self-compassion and resilience training, to improve overall student physical and mental health. Self-compassion was previously reported to reduce self-blame, rumination, catastrophizing, and sleep complaints [54, 55], suggesting that interventions aimed at enhancing self-compassion could mitigate the negative effects of rest intolerance. Our results point to the central role of self-compassion as a mediator in reducing the negative impact of rest intolerance. Considering that self-compassion was found to reduce emotional distress and improve sleep quality, interventions promoting self-kindness and understanding may serve as an effective strategy to counteract the harmful effects of rest intolerance. Specifically, fostering self-compassion in students could help to alleviate the feelings of guilt or discomfort associated with taking breaks, facilitating healthier work-rest cycles, and improving psychological well-being. This aligns with previous findings showing that self-compassion plays a significant role in reducing anxiety, stress, and other negative emotional states (e.g., Fong & Loi, 2016 [46]; Hatun & Kurtça, 2022 [47]), making it a promising avenue for intervention.

Rest intolerance can also be associated with critical psychological factors such as work addiction and burnout. In the contemporary world, work addiction has emerged as a significant concern, with wide-ranging negative implications for individuals. It is characterized by a compulsive engagement in work-related tasks and a persistent cognitive preoccupation with work, often resulting in considerable psychological distress and functional impairment [56]. This maladaptive behavior can adversely impact not only the individual’s well-being but also their interpersonal relationships, particularly with close friends and family. Marked by a loss of control and a chronic nature, work addiction can represent a growing threat to mental health. Consequently, the present study might contribute to the understanding of this phenomenon and provide a foundation for addressing its underlying causes and consequences.

While this study has strengths, several limitations should be acknowledged. First, the cross-sectional design of the current study inherently limits the ability to draw causal inferences or to examine how the relationships among rest intolerance, emotional distress, and sleep complaints evolve over time. Because data were collected at a single point, it is not possible to determine the directionality of effects or to observe potential fluctuations in these variables across different periods. Consequently, the findings should be interpreted as correlational rather than causal. To address these limitations and gain a more comprehensive understanding of the temporal dynamics and underlying causal mechanisms, future research would benefit from employing longitudinal study designs. Such approaches would allow for the tracking of individual changes over time, the identification of potential predictors and outcomes, and the exploration of bidirectional or reciprocal relationships among the key constructs. This would not only strengthen the empirical rigor but also provide more nuanced insights into how rest intolerance interacts with emotional distress and sleep disturbances within diverse populations. Second, all scales used in this study were self-reported, which may have introduced response biases such as social desirability or inaccurate self-assessment, which could have compromised the reliability and validity of the data. Third, the study sample consisted predominantly of female participants (73.5%), which may restrict the generalizability of the findings to the wider population. This gender imbalance could influence the observed relationships among the variables, and thus, caution is warranted when extending the results to more gender-diverse or male-dominant groups. Fourth, while the resilient mindset was found to correlate with emotional distress and sleep complaints, its role in mediating the relationship between rest intolerance and these outcomes was less significant. This suggests that external factors might overshadow the protective effects of resilience in the context of rest intolerance. This finding also highlights the complexity of addressing rest intolerance, as simply cultivating resilience may not be sufficient when pervasive cultural and social pressures, such as competition and comparison, exacerbate the feelings of inadequacy. Future research should take into account other potentially influential variables that were not measured in the present study, as they may impact the relationships examined. For instance, factors such as chronic health conditions, levels of academic stress, and perceived social support could play a significant role in shaping individuals’ experiences of rest intolerance and related psychological outcomes. Including these variables in future studies would provide a more comprehensive understanding of the underlying mechanisms and contextual influences.

In conclusion, this study makes a valuable contribution to previous research on the novel construct of rest intolerance and its relationship with sleep complaints, emotional distress, self-compassion, and resilient mindset. Investigating these variables is particularly crucial in university student populations, as students are closely linked to both well-being and academic outcomes. Our findings convincingly demonstrate the strong negative impact of rest intolerance on emotional distress and sleep quality, while highlighting the significant mediating role of self-compassion in mitigating these adverse effects.

## Data Availability

Data will be available upon reasonable request.

## Declarations

### Pre-registration Statement

This study was not pre-registered.

## References

[1] Li J. Take off the rest-shame syndrome and untie your mind for a better start [Internet]. Red Net; 2023 Dec 13 [cited 2024 May 26]. Available from: https://new.qq.com/rain/a/20231213A061FA00

[2] Wang F, Song H, Meng X, Wang T, Zhang Q, Yu Z, Fan S, Wu Y. Development and validation of the long and short forms of the rest intolerance scale for college students. Pers Individ Dif. 2025;233:112869. 10.1016/j.paid.2024.112869.

[3] Zhang Y. Rejecting the "shame of rest" and returning rest to its essence [Internet]. Red Net; 2023 Dec 14 [cited 2024 May 25]. Available from: https://new.qq.com/rain/a/20231214A09SOX00

[4] Koo HJ. American idle: An examination of leisure guilt, time use, and well-being [doctoral dissertation]. Irvine (CA): University of California; 2023. PQDT:85127228.

[5] Wong B. Why you don’t need to explain your reason for taking a day off [Internet]. HuffPost; 2020 Nov 23 [cited 2024 May 20]. Available from: https://www.huffpost.com/entry/dont-need-explain-reason-taking-day-off_l_5fac30f5c5b635e9de9e4d3a

[6] Granieri A, Franzoi IG, Chung MC. Psychological distress among university students. Front Psychol. 2021;12:647940. 10.3389/fpsyg.2021.647940. 33828513 10.3389/fpsyg.2021.647940 PMC8019774

[7] Mboya IB, John B, Kibopile ES, Mhando L, George J, Ngocho JS. Factors associated with mental distress among undergraduate students in Northern Tanzania. BMC Psychiatry. 2020;20:28. 10.1186/s12888-020-2448-1. 31996200 10.1186/s12888-020-2448-1 PMC6988278

[8] Gardani M, Bradford DR, Russell K, Allan S, Beattie L, Ellis JG, Akram U. A systematic review and meta-analysis of poor sleep, insomnia symptoms and stress in undergraduate students. Sleep Med Rev. 2022;61:101565. 10.1016/j.smrv.2021.101565. 34922108 10.1016/j.smrv.2021.101565

[9] Jiang XL, Zheng XY, Yang J, Ye CP, Chen YY, Zhang ZG, Xiao ZJ. A systematic review of studies on the prevalence of insomnia in university students. Public Health. 2015;129(12):1579–84. 10.1016/j.puhe.2015.07.030. 26298588 10.1016/j.puhe.2015.07.030

[10] Alvaro PK, Roberts RM, Harris JK. A systematic review assessing bidirectionality between sleep disturbances, anxiety, and depression. Sleep. 2013;36(7):1059–68. 10.5665/sleep.2810. 23814343 10.5665/sleep.2810 PMC3669059

[11] Lancel M, Boersma GJ, Kamphuis J. Insomnia disorder and its reciprocal relation with psychopathology. Curr Opin Psychol. 2021;41:34–9. 10.1016/j.copsyc.2021.02.001. 33691218 10.1016/j.copsyc.2021.02.001

[12] Zhou F, Li S, Xu H. Insomnia, sleep duration, and risk of anxiety: a two-sample Mendelian randomization study. J Psychiatr Res. 2022;155:219–25. 10.1016/j.jpsychires.2022.08.012. 36087367 10.1016/j.jpsychires.2022.08.012

[13] Schlarb AA, Claßen M, Hellmann SM, Vögele C, Gulewitsch MD. Sleep and somatic complaints in university students. J Pain Res. 2017;10:1189–99. 10.2147/JPR.S125421. 28572738 10.2147/JPR.S125421 PMC5441659

[14] Grima NA, Bei B, Mansfield D. Insomnia theory and assessment. Aust J Gen Pract. 2019;48(4):193–7. 10.31128/ajgp-12-18-4780. 31256488 10.31128/AJGP-12-18-4780

[15] Nguyen VV, Zainal NH, Newman MG. Why sleep is key: poor sleep quality is a mechanism for the bidirectional relationship between major depressive disorder and generalized anxiety disorder across 18 years. J Anxiety Disord. 2022;90:102601. 10.1016/j.janxdis.2022.102601. 35850001 10.1016/j.janxdis.2022.102601 PMC9945467

[16] Xu X, Elliott B, Peng Y, Jalil D, Zhang W. Help or hindrance? A daily diary study on the workaholism–performance relation. Int J Stress Manag. 2021;28(3):176–85. 10.1037/str0000176.

[17] Luta D, Pogrebtsova E, Provencher Y. The wellbeing implications of thinking about schoolwork during leisure time: a qualitative analysis of Canadian university students’ psychological detachment experiences. J Furth High Educ. 2021;45(6):771–87. 10.1080/0309877X.2020.1813265.

[18] Li X, Malli MA, Cosco TD, Zhou G. The relationship between self-compassion and resilience in the general population: protocol for a systematic review and meta-analysis. JMIR Res Protoc. 2024;13(1):e60154. 10.2196/60154. 39636677 10.2196/60154 PMC11659685

[19] Neff KD. Self-compassion: theory, method, research, and intervention. Annu Rev Psychol. 2023;74(1):193–218. 10.1146/annurev-psych-032420-031047. 35961039 10.1146/annurev-psych-032420-031047

[20] Arslan G. My inner perfectionist and nasty side! Self-compassion, emotional health, and subjective well-being in college students. Pers Individ Dif. 2023;210:112232. 10.1016/j.paid.2023.112232.

[21] Brown L, Houston EE, Amonoo HL, Bryant C. Is self-compassion associated with sleep quality? A meta-analysis. Mindfulness. 2021;12:82–91. 10.1007/s12671-020-01498-0.

[22] De Souza LK, Policarpo D, Hutz CS. Self-compassion and symptoms of stress, anxiety, and depression. Trends Psychol. 2020;28(1):85–98. 10.1007/s43076-020-00018-2.

[23] Raes F. The effect of self-compassion on the development of depression symptoms in a non-clinical sample. Mindfulness. 2011;2:33–6. 10.1007/s12671-011-0040-y.

[24] Ewert C, Vater A, Schröder-Abé M. Self-compassion and coping: A meta-analysis. Mindfulness. 2021;12:1063–77. 10.1007/s12671-020-01563-8.

[25] Munroe M, Al-Refae M, Chan HW, Ferrari M. Using self-compassion to grow in the face of trauma: the role of positive reframing and problem-focused coping strategies. Psychol Trauma. 2022;14(1):157–64. 10.1037/tra0001164. 10.1037/tra0001164 34735189

[26] Fletcher D, Sarkar M. Psychological resilience: a review and critique of definitions, concepts, and theory. Eur Psychol. 2013;18(1):12–23. 10.1027/1016-9040/a00012.

[27] Elliott TR, Perrin PB, Bell AS, Powers MB, Warren AM. Resilience, coping, and distress among healthcare service personnel during the COVID-19 pandemic. BMC Psychiatry. 2021;21:1–12. 10.1186/s12888-021-03506-6. 34615501 10.1186/s12888-021-03506-6 PMC8493044

[28] Liu JJ, Ein N, Gervasio J, Battaion M, Reed M, Vickers K. Comprehensive meta-analysis of resilience interventions. Clin Psychol Rev. 2020;82:101919. 10.1016/j.cpr.2020.101919. 33045528 10.1016/j.cpr.2020.101919

[29] Ang WHD, Lau ST, Cheng LJ, Chew HSJ, Tan JH, Shorey S, Lau Y. Effectiveness of resilience interventions for higher education students: A meta-analysis and metaregression. J Educ Psychol. 2022;114(7):1670–94. 10.1037/edu0000719.

[30] Raes F, Pommier E, Neff KD, Van Gucht D. Construction and factorial validation of a short form of the self-compassion scale. Clin Psychol Psychother. 2011;18(3):250–5. 10.1002/cpp.702. 21584907 10.1002/cpp.702

[31] Arslan G, Wong P. Embracing life’s challenges: developing a tool for assessing resilient mindset in second wave positive psychology. J Happiness Health. 2024;4(1):1–10. 10.47602/johah.v4i1.53.

[32] Allen Gomes A, Ruivo Marques D, Meia-Via AM, Meia-Via M, Tavares J, Fernandes da Silva C, Pinto de Azevedo MH. Basic scale on insomnia complaints and quality of sleep (BaSIQS): reliability, initial validity and normative scores in higher education students. Chronobiol Int. 2015;32(3):428–40. 10.3109/07420528.2014.986681. 25482053 10.3109/07420528.2014.986681

[33] Mıhçıoğlu İ, Malakcıoğlu C, Mutlu HH. Uykusuzluk şikayetleri ve Uyku kalitesi Temel ölçeği’nin Türkçe geçerlilik ve güvenirliği. Turk J Fam Med Prim Care. 2021;15(4):846–52. 10.21763/tjfmpc.971532.

[34] International Test Commission. ITC guidelines for translating and adapting tests. 2nd ed. 2017. https://www.intestcom.org/files/guideline_test_adaptation_2ed.pdf

[35] Hair J, Black WC, Babin BJ, Anderson RE. Multivariate data analysis. 7th ed. Upper Saddle River (NJ): Pearson Educational International; 2010.

[36] Hu LT, Bentler PM. Cutoff criteria for fit indexes in covariance structure analysis: conventional criteria versus new alternatives. Struct Equation Model. 1999;6(1):1–55. 10.1080/10705519909540118.

[37] Hayes AF. Introduction to mediation, moderation, and conditional process analysis: a regression based approach. New York: Guilford; 2018.

[38] Kline RB. Principles and practice of structural equation modeling. 4th ed. New York: Guilford Press; 2015.

[39] Tabachnick BG, Fidell LS. Using multivariate statistics. 6th ed. Boston: Pearson; 2012.

[40] Preacher KJ, Hayes AF. Asymptotic and resampling strategies for assessing and comparing indirect effects in multiple mediator models. Behav Res Methods. 2008;40(3):879–91. 10.3758/BRM.40.3.879. 18697684 10.3758/brm.40.3.879

[41] Miklósi M, Martos T, Szabó M, Kocsis-Bogár K, Forintos D. Cognitive emotion regulation and stress: a multiple mediation approach. Transl Neurosci. 2014;5(1):64–71. 10.2478/s13380-014-0207-9.

[42] Wang F, Bíró É. Determinants of sleep quality in college students: a literature review. Explore (NY). 2021;17(2):170–7. 10.1016/j.explore.2020.11.003. 33246805 10.1016/j.explore.2020.11.003

[43] Yetim U. The impacts of individualism/collectivism, self-esteem, and feeling of mastery on life satisfaction among the Turkish university students and academicians. Soc Indic Res. 2003;61:297–317. 10.1023/A:1021911504113.

[44] Bartlett MJ, Arslan FN, Bankston A, Sarabipour S. Ten simple rules to improve academic work–life balance. PLoS Comput Biol. 2021;17(7):e1009124. 10.1371/journal.pcbi.1009124. 34264932 10.1371/journal.pcbi.1009124 PMC8282063

[45] Tran MAQ, Vo-Thanh T, Soliman M, Ha AT, Van Pham M. Could mindfulness diminish mental health disorders? The serial mediating role of self-compassion and psychological well-being. Curr Psychol. 2024;43(15):13909–22. 10.1007/s12144-022-03421-3. 10.1007/s12144-022-03421-3 PMC9362435 35967505

[46] Fong M, Loi NM. The mediating role of self-compassion in student psychological health. Aust Psychol. 2016;51(6):431–41. 10.1111/ap.12185.

[47] Hatun O, Kurtça TT. Self-compassion, resilience, fear of COVID-19, psychological distress, and psychological well-being among Turkish adults. Curr Psychol. 2022. 10.1007/s12144-022-02824-6. 35345542 10.1007/s12144-022-02824-6 PMC8943103

[48] Rakhimov A, Ong J, Realo A, Tang NK. Being kind to self is being kind to sleep? A structural equation modelling approach evaluating the direct and indirect associations of self-compassion with sleep quality, emotional distress and mental well-being. Curr Psychol. 2023;42(16):14092–105. 10.1007/s12144-021-02661-z.

[49] Hu Y, Wang Y, Sun Y, Arteta-Garcia J, Purol S. Diary study: the protective role of self-compassion on stress-related poor sleep quality. Mindfulness. 2018;9:1931–40. 10.1007/s12671-018-0939-7.

[50] Semenchuk BN, Onchulenko S, Strachan SM. Self-compassion and sleep quality: examining the mediating role of taking a proactive health focus and cognitive emotional regulation strategies. J Health Psychol. 2022;27(10):2435–45. 10.1177/13591053211047148. 34544298 10.1177/13591053211047148 PMC9434207

[51] Akin A, Akin U. Self-compassion as a predictor of social safeness in Turkish university students. Rev Latinoam Psicol. 2015;47(1):43–9. 10.1016/S0120-0534(15)30005-4.

[52] Han J, Jeong S, Hur T, Kim M. How women differently felt guilt from men in korea: focusing on the influence of demographic factors and leisure motivation. Health Care Women Int. 2023;44(1):28–45. 10.1080/07399332.2020.1743995. 32275487 10.1080/07399332.2020.1743995

[53] Hansen J, Hartwell C, Madsen SR. The impact of COVID-19 on Utah women and work: resilient mindset and wellbeing. Res Policy Brief. 2021;36:1–5. https://digitalcommons.usu.edu/marketing_facpub/139/.

[54] Amaral AP, Soares MJ, Pinto AM, Pereira AT, Madeira N, Bos SC, Marques M, Roque C, Macedo A. Sleep difficulties in college students: the role of stress, affect and cognitive processes. Psychiatry Res. 2018;260:331–7. 10.1016/j.psychres.2017.11.072. 29227897 10.1016/j.psychres.2017.11.072

[55] Hamrick LA, Owens GP. Exploring the mediating role of self-blame and coping in the relationships between self‐compassion and distress in females following the sexual assault. J Clin Psychol. 2019;75(4):766–79. 10.1002/jclp.22730. 30552686 10.1002/jclp.22730

[56] Atroszko PA, Demetrovics Z, Griffiths MD. Work addiction, obsessive-compulsive personality disorder, burn-out, and global burden of disease. Implications from the ICD-11. Int J Environ Res Public Health. 2020;17(2):660. 10.3390/ijerph17020660. 10.3390/ijerph17020660 PMC7014139 31968540

